# Annual acknowledgement of manuscript reviewers

**DOI:** 10.1186/1475-2840-12-43

**Published:** 2013-03-27

**Authors:** Alexander Tenenbaum, Enrique Fisman

**Affiliations:** 1 Cardiac Rehabilitation Institute, Sheba Medical Center, Tel-Hashomer, 52621, Israel; 2 Sackler Faculty of Medicine, Tel-Aviv University, Tel-Aviv, 69978, Israel

## Abstract

**Contributing reviewers:**

The Editors of Cardiovascular Diabetology wish to thank all reviewers, both Editorial Board Members and external referees, who contributed to the journal in 2012 (Volume 11), and whose enthusiastic support in providing the essential service of peer review continues to improve the scientific quality of the journal and enable its goals to be achieved.

## 

Mehrshad Abbasi

Iran

Pasquale Abete

Italy

Hitoshi Adachi

Japan

Carlos A. Aguilar Salinas

Mexico

Aila Ahola

Finland

Yoshifusa Aizawa

Japan

Yasuo Akanuma

Japan

Rajadurai Akilen

United Kingdom

Nasser Al-Daghri

Saudi Arabia

Nikolaos Alexopoulos

Greece

Zaid Hezam Al-Hamodi

Malaysia

Mouaz Al-Mallah

Saudi Arabia

Amy Alman

United States of America

Alexandra Alvarsson

Sweden

Tetsuya Amano

Japan

Giuseppe Ambrosio

Italy

Charlotte Andersson

Denmark

Christian Anderwald

Austria

Ioanna Andreadou

Greece

Daniele Andreini

Italy

Francesco Andreozzi

Italy

Monica Andrews

Chile

Hidenori Arai

Japan

Hisatomi Arima

Australia

Benoit Arsenault

Netherlands

Kei Asayama

Belgium

Yoshimasa Aso

Japan

Kirsi Auro

Finland

Pablo Avanzas

Spain

Vajreswari Ayyalasomayajula

India

Gopal Babu

United States of America

Ljuba Bacharova

Slovakia

Richard Bagnall

United Kingdom

Christopher Baines

United States of America

Enzo Ballotta

Italy

Yongping Bao

United Kingdom

Elizabeth Barrett-Connor

United States of America

Rene Baudrand

Chile

John Baynes

United States of America

Abdulbari Bener

Qatar

Don Benjamin

Switzerland

Joline Beulens

Netherlands

Michel Beylot

France

Dwaipayan Bharadwaj

India

Yochai Birnbaum

United States of America

Simona Bo

Italy

Mona Boaz

Israel

Leandro Boer-Martins

Brazil

Steffen Bohler

Germany

L Cornelius Bollheimer

Germany

Fabrice Bonnet

France

Carlo Briguori

Italy

Carlos Brotons

Spain

Christa Buechler

Germany

Clay Bunn

United States of America

Monika Buraczynska

Poland

Christof Burgdorf

Germany

Francesco Cacciatore

Italy

Stefano Cagnin

Italy

Maria Calatayud

Spain

Duncan Campbell

Australia

Massimiliano Caprio

Italy

Françoise Carlotti

Netherlands

Axel Carlsson

Sweden

Jan Cederholm

Sweden

Piero Ceriana

Italy

Rajesh Chaudhary

Thailand

Wei Chen

United States of America

Mien-Cheng Chen

Taiwan

Jaw-Wen Chen

Taiwan

Hsing I Chen

Taiwan

Brian Chen

United States of America

Shiyou Chen

United States of America

Min Cheng

China

Hoi Lun Cheng

Australia

Juei-Tang Cheng

Taiwan

Adnan Chhatriwalla

United States of America

Robert Chilton

United States of America

Jaye Chin-Dusting

Australia

Tak-Shing Ching

Taiwan

Sungsam Cho

Japan

Zhao Zhong Chong

United States of America

Nain-Feng Chu

Taiwan

Peter Chudý

Slovakia

Arrigo FG Cicero

Italy

Iwona Cicha

Germany

Emmanuel Cosson

France

Stuart Craig

United States of America

Cora Cymeryng

Argentina

Wangde Dai

United States of America

Andreas Daiber

Germany

Robert Dalla Pozza

Germany

Paresh Dandona

United States of America

Merav Darash-Yahana

Israel

Michael Dashwood

United Kingdom

Wendy Davis

Australia

Dirk De Bacquer

Belgium

Peter Wilhelmus de Leeuw

Netherlands

Richard Dearborn Jr.

United States of America

Christi Deaton

United Kingdom

Mark DeBoer

United States of America

M Isabel del Olmo

Spain

Aldona Dembinska-Kiec

Poland

Patrick Dessein

South Africa

George Dimitriadis

Greece

Hong Ding

Qatar

Ellen Dirkx

Belgium

Dragan Djuric

Serbia

Sergio Dubner

Argentina

Reinhard Duehlmeier

Germany

Jacques Dumont

Belgium

Kensuke Egashira

Japan

Kai Eggers

Sweden

Robert Elkeles

United Kingdom

Jackie Elliott

United Kingdom

Mehmet Erdogan

Turkey

Serpil Eroglu

Turkey

David Faeh

Switzerland

Giuseppe Faggian

Italy

Inês Falcão-Pires

Portugal

Britt Falskov

Denmark

Csaba Farsang

Hungary

John Feiner

United States of America

Trevor Ferguson

Jamaica

Michiaki Fukui

Japan

Tohru Funahashi

Japan

Martha Funnell

United States of America

Masato Furuhashi

Japan

Trudy Gaillard

United States of America

Xin Gao

China

Luis García-Ortiz

Spain

William Garver

United States of America

Irene Gazi

Greece

Zhi-Dong Ge

United States of America

Adriana Georgescu

Romania

Cosimo Giannini

United States of America

Bruna Gigante

Sweden

Janaina Gonçalved

Brazil

Jørgen Gravning

Norway

Jacoba P Greving

Netherlands

Garrett Gross

United States of America

Bruno Guerci

France

Zhixin Guo

China

Hua Guo

United States of America

Alok Kumar Gupta

United States of America

Alev Gurgun

Turkey

Matthew Gurka

United States of America

Ida Gustafsson

Denmark

Anne Hafstad

Norway

Ganesh Halade

United States of America

Samar M. Hammad

United States of America

Juying Han

Canada

Scott Harding

United Kingdom

Fatma Harzallah

Tunisia

Goji Hasegawa

Japan

Yuichi Hattori

Japan

Toshio Hayashi

Japan

Zeliha Hekimsoy

Turkey

Hans-Werner Hense

Germany

Michel P Hermans

Belgium

Ramon Hermida

Spain

Hiroshi Hirose

Japan

Marion Hofmann

United States of America

Martin Hohenegger

Austria

Philip Home

United Kingdom

Dongming Hou

United States of America

Peng Hu

China

Qiaobing Huang

China

Yu Huang

China

Abdelaziz Hussein

Egypt

Chii-Min Hwu

Taiwan

Iskandar Idris

United Kingdom

Satoshi Ikeda

Japan

Ignatios Ikonomidis

Greece

Wilfred Ip

Canada

Masaharu Ishihara

Japan

Yasuhiro Ishihara

Japan

Nobukazu Ishizaka

Japan

Nobuhiko Ishizuka

Japan

Milosz Jaguszewski

Switzerland

Karin Jandeleit-Dahm

Australia

Myung Ho Jeong

Korea, South

Gyorgy Jermendy

Hungary

Lin Jiang

United States of America

Manuel Jimenez-Navarro

Spain

Odd Erik Johansen

Norway

Brent Johnston

Canada

Michael Joner

Germany

Chan Hee Jung

South Korea

Markus Juonala

Finland

Satomi Kagota

Japan

Radhika Kakarala

United States of America

Mohd Fozi Kamarudin

Malaysia

Masahiro Kamouchi

Japan

Ippei Kanazawa

Japan

Raj Kandpal

United States of America

Hideaki Kaneto

Japan

Tom Karagiannis

Australia

Jens Kastrup

Denmark

Naoto Katakami

Japan

Akihiko Kato

Japan

Ryuichi Kawamoto

Japan

Malte Kelm

Germany

Mykola Khalangot

Ukraine

Haseeb Khan

Saudi Arabia

Zia Khan

Canada

Ahsan Khandoker

Australia

Madhu Khullar

India

Daejung Kim

South Korea

Shokei Kim-Mitsuyama

Japan

Klaus-Dieter Kohnert

Germany

Pappachan Kolattukudy

United States of America

Malcolm Koo

Canada

Magdalena Kostkiewicz

Poland

Masoumeh Kourosh Arami

Japan

Irina Kowalska

Poland

Detlef Kozian

Germany

Marek Kozinski

Poland

Rungroj Krittayaphong

Thailand

Wiktor Kuliczkowski

Poland

Hemant Kulkarni

United States of America

Adnan Kurdal

Turkey

Claudia Kusmic

Italy

Johanna Kuusisto

Finland

Andreas Kuznik

United States of America

Veronique Lacombe

United States of America

Jorma Lahtela

Finland

Tomi Laitinen

Finland

Wendy Lane

United States of America

Cia-Hin Lau

Malaysia

Harold Lazar

United States of America

Tennille Leak-Johnson

United States of America

Verna Lee

Malaysia

Won Young Lee

Korea, South

Byung-Wan Lee

Korea, South

Yong-Jae Lee

Korea, South

I-Te Lee

Taiwan

Michael Lehrke

Germany

Ioannis Lekakis

Greece

Konstantinos Letsas

Greece

Andrew Levy

Israel

Kae-Woei Liang

Taiwan

Wei Liang

China

Yi-Chu Liao

Taiwan

Atip Likidlilid

Thailand

Kuan Pin Lin

Taiwan

Michael Lipinski

United States of America

Que Liu

United States of America

Bicheng Liu

China

Brian Bridal Løgstrup

Denmark

Gary Lopaschuk

Canada

Carolina Lourenço

Portugal

Hongyun Lu

China

Stephan Lüders

Germany

Olga Lunegova

Kyrgyzstan

Christoph Maack

Germany

Imad Maatouk

Germany

Stephen Macdonald

Australia

Rogelio Alberto Machado

Argentina

Agnieszka Madro

Poland

Shiro Maeda

Japan

Sujata Mahadik

India

Atheline Major-Pedersen

Denmark

Safarina Golfiani Malik

Indonesia

Kai Mao

United States of America

Pedro J Marín

Spain

Raffaele Marfella

Italy

Jennifer Martin

Australia

Francisco Javier Martinez-Martin

Spain

Raul Martins

Portugal

Masafumi Matsuda

Japan

Laetitia Mauge

France

Helen McDonald

United Kingdom

Jane McElroy

United States of America

Philip McTernan

United Kingdom

Manoj Medal

India

Patrick Meimoun

France

Christa Meisinger

Germany

Jordi Merino

Spain

Patricia Metcalf

New Zealand

Takayuki Miki

Japan

Masoud Mirzaei

Australia

Toru Miyoshi

Japan

Denise Momesso

Brazil

Louis Monnier

France

Monica Montagnani

Italy

Ryuichi Morishita

Japan

Yusuke Moritoh

Japan

Antonio Muscari

Italy

Elza Muscelli

Italy

Fawaz Mzayek

United States of America

Sath Nag

United Kingdom

Shashidhar Nagaraj

India

Kei Nakajima

Japan

Manouchehr Nakhjavani

Iran

Emilio Nardi

Italy

Loredan Stefan Niculescu

Romania

Peter Nilsson

Sweden

Peter Nilsson

Sweden

Koichi Node

Japan

Ryuji Nohara

Japan

Anna Norhammar

Sweden

Mads Nybo

Denmark

Anders Nygren

Canada

Mark O'Donnell

United Kingdom

Hiroshi Ogawa

Japan

Hisao Ogawa

Japan

Robert Ohsfeldt

United States of America

Yosuke Okada

Japan

Satoshi Okayama

Japan

Sukma Oktavianthi

Indonesia

Silvio Oliveira Junior

Brazil

Trine Baur Opstad

Norway

Emilio Ortega

Spain

Alfonso Otero Gonzalez

Spain

Noriyuki Ouchi

Japan

Nobuaki Ozaki

Japan

Pal Pacher

United States of America

Raluca Pais

France

Barry Palmer

New Zealand

Yang Pan

United States of America

Dimitrios Papadopoulos

Greece

Ioannis Papassotiriou

Greece

Athanasios G. Papavassiliou

Greece

Cheol-Young Park

South Korea

Hyeong-Kyu Park

South Korea

Joong-Yeol Park

South Korea

Anthony Passerini

United States of America

Ulrik Pedersen-Bjergaard

Denmark

Michael Pencina

United States of America

Wenhui Peng

China

Grant Pierce

Canada

Antonio Pinto

Italy

Dario Pitocco

Italy

Bertram Pitt

United States of America

Michael Poteser

Austria

Fabrice Prunier

France

Tran Quang Binh

Vietnam

Edward Randell

Canada

Ankit Rathod

United States of America

Itamar Raz

Israel

Peter Reaven

United States of America

Flavio Reis

Portugal

Alan Remaley

United States of America

Jun Ren

United States of America

Markus Resch

Germany

Fausto Rigo

Italy

Stefano Rimoldi

Switzerland

Maria Rosaria Rizzo

Italy

Testa Roberto

Italy

Bruno Rodrigues

Brazil

Martha Rodriguez-Moran

Mexico

Robert Rosenson

United States of America

Ina-Maria Rückert

Germany

Sandeep Saha

United States of America

M Ram Sairam

Canada

Masaya Sakamoto

Japan

Naoki Sakane

Japan

Juha Saltevo

Finland

Rafael Salto

Spain

Erik Salum

Estonia

Paulo Santos

Brazil

Gaetano Santulli

United States of America

Ayhan Saritas

Turkey

Kevin Schalinske

United States of America

Arthur Scholte

Netherlands

Aletta Schutte

South Africa

Karl Schwab

Germany

Neal Scott

United States of America

Fernando Segade

United States of America

Yutaka Seino

Japan

Ken-ichi Serizawa

Japan

Amir Shafat

Ireland

Michal Shani

Israel

Jayashree Shanker

India

Sushma Sharma

United States of America

Sunil sharma

India

Michael Shechter

Israel

Weifeng Shen

China

Wayne Sheu

Taiwan

Zumin Shi

Australia

Rei Shibata

Japan

JaeYong Shim

Korea, South

Toshio Shimada

Japan

Kazunori Shimada

Japan

Tetsuro Shishido

Japan

Manisha Shrivastav

United States of America

David Simmons

United Kingdom

Scot Simpson

Canada

Vivek Singh

United States of America

Susan Sisson

United States of America

Sandhya Sitasawad

India

Ivonne Sluijs

Netherlands

Irina Smirnova

United States of America

Andries Smit

Netherlands

Janet Snell-Bergeon

United States of America

Elsayed Z. Soliman

United States of America

Maria de Fatima Sonati

Brazil

Dinesh Kumar Srinivasan

Singapore

Eberhard Standl

Germany

Arie Steinvil

Israel

Stephanie Stock

Germany

Joshua Stolker

United States of America

Celia Strunz

Brazil

Milton Suarez-Ortegón

Colombia

Elena Succurro

Italy

Takayoshi Suganami

Japan

J David Symons

United States of America

Cheuk Chun Szeto

Hong Kong

Yasuharu Tabara

Japan

Heinrich Taegtmeyer

United States of America

Isao Taguchi

Japan

Julio Takada

Brazil

Yasuo Takahashi

Japan

Takeshi Takami

Japan

Kumiko Takemori

Japan

Junichi Taki

Japan

Charmaine Tam

Australia

Luosheng Tang

China

Giovanni Targher

Italy

Ulrich Tebbe

Germany

Alexander Tenenbaum

Israel

Nicholas Tentolouris

Greece

Vicki Thallas-Bonke

Australia

Stine Thomsen

Denmark

Stephen Thomson

United States of America

Chengju Tian

United States of America

Ivan Tkac

Slovakia

Hirofumi Tomiyama

Japan

Arpad Tosaki

Hungary

Dimitris Tousoulis

Greece

Antonino Tuttolomondo

Italy

Shiro Uemura

Japan

Matti Uusitupa

Finland

Rodolfo Valdez

United States of America

Serafina Valente

Italy

Charissa van den Brom

Netherlands

Ingrid van der Mei

Australia

Mariska van Vliet

Netherlands

Luca Vanella

Italy

Keri Vartanian

United States of America

Elena Velkoska

Australia

Marianne Verhaar

Netherlands

Suman Verma

India

Richard Verrier

United States of America

Saula Vigili de Kreutzenberg

Italy

Anders Vik

Norway

Jeffrey Voigt

United States of America

Adriaan Voors

Netherlands

Christina Voulgari

Greece

Brian Walker

United Kingdom

Yujie Wang

United States of America

Xin Wang

United Kingdom

Wei Wang

United States of America

Wuan-Szu Wang

Taiwan

Marie-Louise Ward

New Zealand

G. Russell Warnick

United States of America

Anna Watson

Australia

Julian Widder

Germany

Robert Wilensky

United States of America

Jenifer Wogen

United States of America

Cheuk-Kit Wong

New Zealand

Wen-Bin Wu

Taiwan

Yi Wu

United States of America

Xunde Xian

United States of America

Liang Xiao

China

Huang Xienan

China

Ya-Wei Xu

Colombia

Shiming Xu

United States of America

Susan Xu

United States of America

Biao Xu

China

Daisuke Yabe

Japan

Neerja Yadav

India

Kazuhiro Yamamoto

Japan

Koichi Yamamoto

Japan

Yi-Ling Yang

Taiwan

Lina Yang

United States of America

Zhen Yang

China

Bu Yeap

Australia

Joseph Yeboah

United States of America

Fawn Yeh

United States of America

Kai Hang Yiu

Hong Kong

Hiroshi Yoshida

Japan

Xinhua Yu

United States of America

Han-Gang Yu

United States of America

Markus Zabel

Germany

Feng Zhang

United States of America

Giacomo Zoppini

Italy

Yunzeng Zou

China

Chenhui Zou

United States of America

Giovanni Zuliani

Italy

